# The impact of Danmaku-based and synchronous peer feedback on L2 oral performance: A mixed-method investigation

**DOI:** 10.1371/journal.pone.0284843

**Published:** 2023-04-25

**Authors:** Hualing Gong, Da Yan

**Affiliations:** School of Foreign Languages, Xinyang Agriculture and Forestry University, Xinyang, China; Ahvaz Jundishapur University: Ahvaz Jondishapour University of Medical Sciences, ISLAMIC REPUBLIC OF IRAN

## Abstract

Advancement of research in education has propelled the augmentation of theoretical and practical knowledge in learning-oriented feedback. In recent years, the channels, modes, and orientations of feedback became manifold. Copious empirical evidence from the body of literature supported the strength of feedback in enhancing learning outcomes and promoting the motivation of learners. However, compared to the popularity in implementation and fruitfulness of findings in other educational domains, the application of state-of-the-art technology-enhanced feedback in fostering students’ L2 oral abilities remain few and far between. To address the knowledge gap, the present study endeavored to investigate the effect of Danmaku-based and synchronous peer feedback on L2 oral performance and the acceptance thereof among students. Adopting a mixed-method design, the study recruited 74 (n = 74) undergraduate English majors from a Chinese university for a 16-week 2x2 experiment. The collected data were analyzed through statistical and thematic analysis respectively. The findings revealed that Danmaku-based and synchronous peer feed-back was impactful on students’ performance in L2 oral production. Furthermore, the impacts of peer feedback on subdomains of L2 competence were statistically analyzed. Regarding students’ perceptions, the incorporation of peer feedback was generally favored by participants who were satisfied and motivated in the learning process but lacked confidence in their assessment literacy. Furthermore, students expressed their agreement with the benefit of reflective learning and the subsequent enrichment in knowledge and horizon. The research was significant for its conceptual and practical contribution for follow-up researchers and educators in L2 education and learning-oriented feedback.

## 1 Introduction

With the development of education, the purpose and orientation of assessment and feedback evolved gradually [1,2]. The conceptual change could be observed alongside with the popularity to adopt strategies of assessment and feedback in pedagogy for improvement of teaching and learning [3]. Hattie and Timperley [4] defined feedback as information provided by an individual about his/her performance and relevant perception. With the emergence of learning-oriented assessment, e.g., formative assessment and “assessment for learning”, the interdependence between these assessment and feedback was further articulated [5]. Feedback was believed to be an integral part of formative assessment, a broader framework focusing on information gathering and provision for improvement of educational quality [6]. In an early yet seminal work, Kluger and DeNisi [7] argued that the response to and action taken on feedback were more significant than the types of feedback received. In a similar vein, Lui and Andrade [8] asserted that the connotation of feedback has shifted from “giving” information to “receiving” information. Specifically, Liu and Andrade [8] disintegrated the internal process of feedback into a four-step procedure: 1) initial motivation; 2) elicitation; 3) interpretation of feedback and 4) decision-making based on feedback. Scholars also emphasized “feedback culture”, with which students would be encouraged to participate in feedback to improve learning outcomes [9].

In practice, educators and researchers have make major headways in developing, implementing, incorporating learning-oriented feedback in pedagogy. First, several different types of feedback were actively practiced, e.g., reinforcement/punishment feedback, corrective feedback and high-information feedback [10]. For example, corrective feedback has been widely used in language learning, e.g., EFL classrooms [11] and translation training [12], etc. Second, the channels of feedback were manifold. According to the body of literature, three major types of feedback were applied, e.g., oral, written and technology-enhanced [10]. Regarding technology-enhanced feedback, the technologies adopted included video, audio and computer programs based on natural language processing [13,14] or audio recognition [15]. Third, the directions of feedback included teacher feedback, students’ feedback and peer feedback. The typology in the directions of feedback was in tandem with the arguments advocated by the school of formative assessment researchers that multiple agents were involved in the process of teaching and learning, e.g., instructor/teacher, students as learners and students as peer learners [16,17].

The popularity of applying feedback in education could be attributed to the asserted strength of the effects of feedback on education. According to the findings from the review by Kluger and DeNisi, an average effect of 0.38 of feedback was presented based on the synthetization of 131 studies with more than 10,000 participants [7]. However, the effect size observed by Kluger and DeNisi was challenged by follow-up researchers since approximately one-third among the reviewed cases in their study produced negative effects [10]. In accordance with the results from the successive meta-analyses, the effect size was recalibrated to a relatively higher level between 0.70 and 0.79 [18,19]. Based on the effect size from documented cases, the impact of peer feedback on general academic achievement of learners has been widely accepted [10].

With the advancement in our understanding pertaining to the effects of peer feedback in language education, headways were made in utilizing multiple sources of feedback to enhance learning, including state-of-the-art technologies [15]. At the same time, orientations, timing and modes of feedback were systematically investigated in the plethora of literature [20,21]. However, there existed a knowledge gap in understanding the interactive effects of feedback modes, e.g., the timing of feedback and technology-enhanced feedback channels, e.g., interactive and video-based feedback. The lack of research in the specific field limited our growing understanding in the nature, mechanism, effectiveness and strategies of peer feedback in a modern language learning context.

For the present study, the aim was to examine the effects of various types of feedback on students’ learning achievement and their reflections thereof. Since the research was contextualized in L2 oral teaching and peer feedback, existing literature regarding the key variables and concepts of the present study were reviewed in the following sections.

The study was significant both practically and theoretically. First, the adoption of Danmaku as a feedback channel was rarely practiced in the context of language education. The study served as a pilot evaluation of the feasibility, affordance and the effects of this innovative feedback practice. Second, Danmaku-based peer feedback was grounded on a sociocultural theoretical perspective of learning. As a result, the innovative endeavor conducted in the study was expected to produce generalizable outcomes for relevant studies in the field, e.g., computer-mediated peer feedback, technology-enhanced language learning, etc. Third, the interactive effects of the timing of feedback and the channels or modes of feedback remained less frequently examined. The outcomes and findings from the present study would contribute to expand our understanding in the field.

### 1.1 Peer feedback

As one the major directions of feedback, peer feedback was argued to be able to enhance both academic skills, reflective abilities and collaborative interaction among students [22]. Based on the review of 24 quantitative studies, Huisman et la. [23] argued that the effects of peer feedback resulted in better improvement in learning outcomes than self-assessment and learners without any feedback. Similar results could be observed from abundant documents, e.g., implementing peer feedback in a self-regulated learning environment [24], incorporating peer feedback in interpreter training curricular [25,26], and using peer feed to promote second language acquisition [27]. Additionally, studies asserted that peer feedback was beneficial to students’ psychological wellbeing and development of motivation [28–30]. The relationship between motivation and peer feedback could be understood from multiple perspectives, i.e., peer feedback functioned both as a medium to reflect motivation [31] and a measure to enhance motivation [32]. In practice, researchers have tried to incorporate advancement from multiple disciplines to augment the efficacy of peer feedback, e.g., using blog as a medium of peer feedback [33], including chatbot as an alternative source of peer feedback [34], etc. Noticeably, unitary strategy for peer feedback should be rejected as students from different countries tended to behave differently in feedback [31]. Apart from the empirical research on the effectiveness of peer feedback on learning outcomes and learning motivation, scholars paid substantial attention to the strength of peer feedback in promoting students’ uptake [35] and assessment literacy [36,37]. In a study on the peer evaluation of feedback, the effects of peer feedback on the cultivation of assessment literacy was manifested and testified [38].

In reality, contradiction could be observed regarding the relatively limited abilities of students to produce actionable peer feedback. The reason could be attributed to the fact that students were not well-established in assessment literacy, which was generally not included as an educational objective [36,37]. In an empirical study lasted for two years, researchers merited the quality and characteristics of peer feedback based on their investigation through blind review [22]. However, as observed by many researchers, the quality of peer feedback was relatively not satisfactory [39,40]. The contradiction could be mediated with the findings from a few studies that the lack of quality in peer feedback would be significantly improved, if substantial training was offered [41], enough space of experimentation was granted [42], well-designed procedures and credible instrument were offered [43], and state-of-the-art technologies were adopted [44]. Interestingly, the issues encountered in studies with unsatisfactory quality of peer feedback generally attributed the underlying reason to students’ limited assessment literacy, in turn the practice of peer feedback was believed to be highly effective in promoting such ability [36–38].

In a nutshell, the development and implementation of peer feedback was endorsed by abundant evidence from existing literature. Additionally, educators and researchers have made major advancement to usher in alternative and innovative modes of feedback that were tailored to suit the needs of domain-specific demands in actual educational settings.

### 1.2 Technology-enhanced channels of feedback

As identified by Wisniewsk et al. [10] in their review, feedback could be conducted and presented through multiple channels, e.g., written, oral, audio/video-based and computer-assisted. With the development of technology, the application of technology-enhanced resources/tools surged. In the study, technology-enhanced feedback was operationally defined as using audio/video-based and computer-assisted feedback in education.

Audio/video-based Feedback (AVBF) has been widely used in multiple educational settings. As asserted in a study to implement AVBF in pre-service teacher training, the researcher argued that AVBF would add value to the educational environment [45]. In the study, the finding revealed that students from a AVBF group would contribute feedback in greater detail in addition to a higher level of perceived feedback competence [45]. In a similar fashion, implementation of AVBF in other educational or training context received positive effects on academic achievement or training outcomes, e.g., using AVBF to foster elite players [46], competent surgical talents [47], talents with communication skills [48]. Additionally, a key strength of AVBF was its reproducible nature [49]. Recorded in video and/or audio, the performance of a student or a trainee could be retrieved and reviewed during and after the peer feedback process.

In recent years, a growing number of studies incorporating state-of-the-art information communication technologies (ICT) emerged. For example, automatic feedback were provided to assist teaching and learning in a variety of educational settings, e.g., L2 writing [50], computer science [51], online learning environment [14], and early childhood education [52]. Researchers and educators have attempted to incorporate many technologies to develop new modes of learning-oriented feedback, e.g., augmented reality [53], natural language processing [54], machine learning algorithms [55], etc. However, the attempts were generally few and far between. The paucity of systematic studies and well-designed implementation constrained the advancement in technology-enhanced feedback.

Among all available channels for learning-oriented feedback, the potential of Danmaku was piloted by a few precedent studies. Danmaku, also known as bullet curtain **[弹幕** or **だんまく]**, referred to the interactive comments in the form of synchronized subtitles posted by viewers of video playback or livestreaming [56]. In a study to investigate students’ interaction in MOOC, the researchers argued that the incorporation of Danmaku in MOOC resulted in better results than conventional MOOC learning model (i.e., video-based learning + forum for discussion) [57]. Similar results could be found in studies situated in different context [56,58]. However, the application of Danmaku as a channel for learning-oriented feedback remain insufficiently studied and reported.

In the present study, the conceptualization and typology of the technology-enhanced channels of peer feedback were fundamental to develop and implement peer feedback strategies that could be applied to enhance learning outcomes and experiences in L2 oral English courses.

### 1.3 Timing of feedback

Regarding the timing of feedback, two major types of feedback were identified: synchronous and asynchronous feedback. In practice, both two types of feedback were widely used in education settings. However, empirical evidence pointed to contradictory arguments on the effectiveness of asynchronous or synchronous feedback. For example, a comparative study contextualized in EFL writing education revealed that despite the satisfaction of learners towards both two types of feedback, asynchronous feedback was more usable than its counterpart [20]. Reversely, the results from another comparison between asynchronous feedback and synchronous feedback asserted that the synchronous feedback was more impactful on the improvement of learners’ writing accuracy in writing [21].

Like other feedback strategies, both synchronous and asynchronous feedback would contribute to the improvement of students’ learning motivation. In a recent study conducted during the outbreak of Covid-19, a group of researchers found that students within a synchronous setting reported greater support experienced for the fulfillment of their basic psychological needs [59]. However, synchronous feedback was not a silver bullet to motivate learners. In a study examine the effects of electronic feedback, the researcher found that synchronous feedback risked of posing extra psychological burden among some respondents [60].

For its alleged strength in promoting academic achievement and learning motivation, the interest to implement synchronous feedback in actual educational settings emerged in recent years. However, for language education, the utilization of synchronous feedback concentrated on EFL writing [20]. For the differences between effects of synchronous feedback or asynchronous feedback on L2 oral production competence, relevant researcher remained few and far between, e.g., an initial effort to use mobile applications for L2 proficiency [61], and a study bearing much resemblance using computer-assisted tools [62]. Most existing research focused on the application of computer-mediated communication (CMC), which was essentially text chat tools used in classrooms. Nonetheless, the existing literature were either outdated or in shortage of quantitative evidence to examine the variances in effects between the two modes of peer feedback. Consequently, for the contemporary era, we faced a paucity in research examining the effects of feedback with different timing. In the present study, the word “timing” was used to denote the synchronism of different types of feedback.

### 1.4 The study

Against the above backdrop, the present study set out to investigate the impact of Danmaku-based and synchronous peer feedback on the performance of learners’ L2 oral production. To achieve the research objective, the following questions would be answered:

**RQ1:** To what extent does Danmaku-based and synchronous feedback impact on student’s performance in L2 oral production?
**RQ2:** How do students perceive and evaluate the application of Danmaku-based and synchronous peer feedback in L2 oral English courses?

## 2. Materials and methods

### 2.1 Design

The present study adopted a mixed-method design to examine the impact of Danmaku-based synchronized feedback on learner’s L2 oral performance. Specifically, a convergent parallel design was adopted [63]. The convergent parallel design refers to the simultaneous collection of both qualitative and quantitative data before a holistic comparison or triangulation to produce comprehensive interpretation of the results and findings [64]. The rationale for the choice of the convergent parallel design was to triangulate both the qualitative and quantitative data collected to bring about in-depth findings complimentarily. In the present study, students’ experiences and reflections towards Danmaku-based and synchronous feedback were collected and analyzed as qualitative data; while their performances in a summative test measuring their L2 oral performance were collected and examined as quantitative data.

### 2.2 Context and participants

The present study took place in an undergraduate university in China. At present, there were a total of 465 students (N = 465) as learners in a Business English program. For students in the undergraduate programs, two-semester L2 oral English courses were mandatory.

Two intact classes of freshmen with a total of 74 students (n = 74) were selected from the population through cluster random sampling. By adopting the cluster random sampling method, the population was first divided into smaller non-overlapping subpopulations, also known as clusters. Then some of the clusters were randomly selected as samples of the study. The reason for the cluster random sampling method was to keep alignment with normal order of pedagogical activities. All participants were Chinese with an average age of 19.3 years. Students’ scores in a placement test administered after the admission was provided to represent their overall competence in the English language. The placement test is designed based on existing and reliable language ability and aptitude tests, i.e., the LLAMA tests [65], and the accredited College English Test in China [66]. The demographical information of the sample was shown in Table 1.

**Table 1:** Demographical information of participants.

Classes	Gender	English Grade in CEPT^1^	
Class #1	Male: 15 (39.5%) Female: 23 (60.5%)	<60	4 (10.53%)
		61–70	16 (42.11%)
		71–80	11 (28.95%)
		81–90	6 (15.79%)
		>91	1 (2.63%)
Class #2	Male: 10 (27.8%) Female: 26 (72.2%)	<60	2 (5.56%)
		61–70	13 (36.11%)
		71–80	10 (27.78%)
		81–90	8 (22.22%)
		>91	3 (8.33%)

^1^ CEPT: A post-admission placement test named College Entrance Placement Test. The test is designed individually for each program in the university. For the case of the EFL learners in the study, the contents of the test were oriented to holistically assess students’ language aptitude and abilities.

### 2.3 The modes of feedback

Being a study to investigate the impact of Danmaku-based and synchronous feedback on student’s performance of L2 oral production, the present study adopted a comparative stance to study the effect of the following modes of feedback in learning Oral English. The feedback adopted different channels (i.e., written/oral and Danmaku-based) and timing (i.e., synchronous and asynchronous) for feedback.

#### Danmaku-based and synchronous feedback

**Fig 1:**
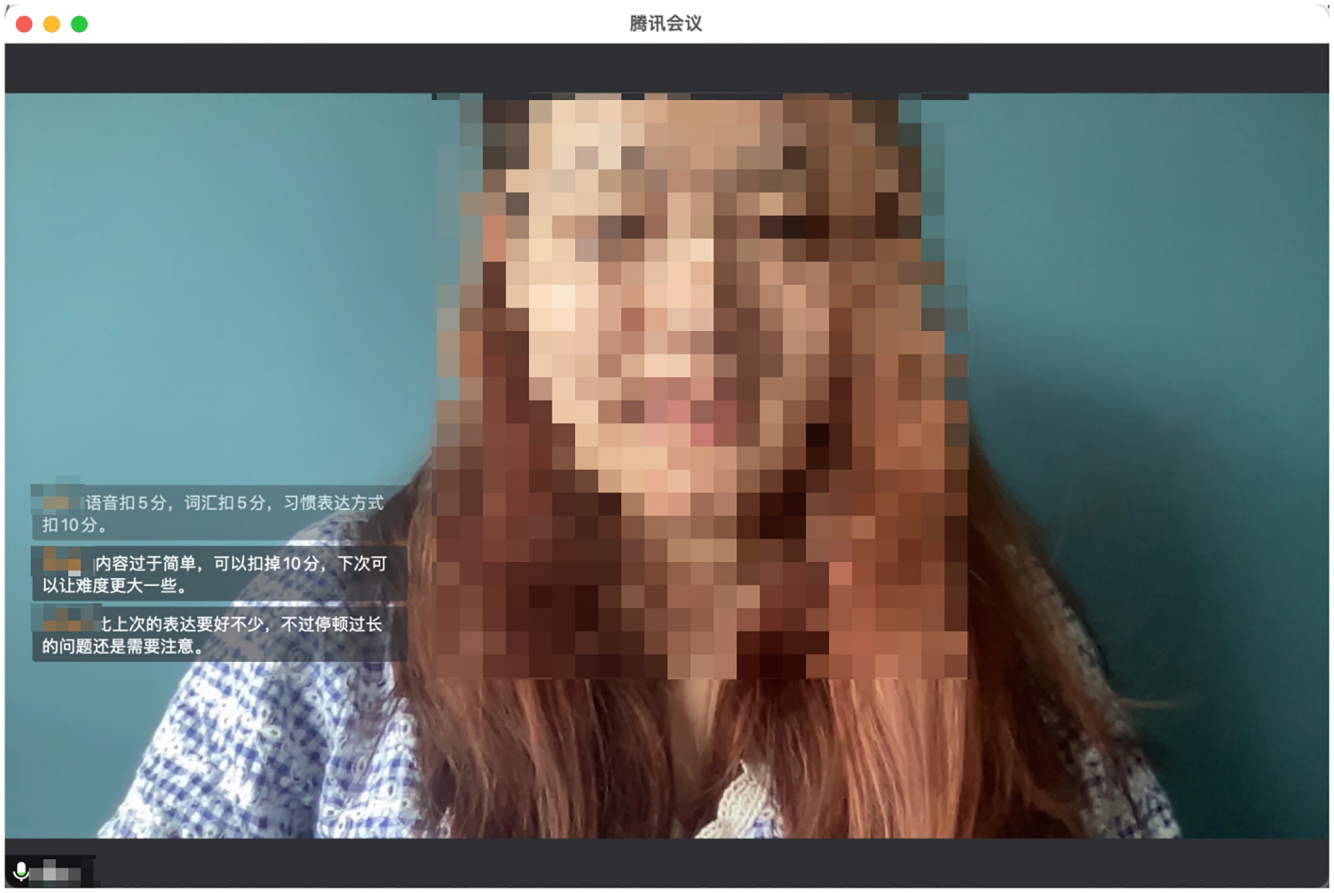
Screenshot of a session of L2 oral English with Danmaku-based and synchronous feedback comments posted by peer students. Note: Names and faces of students were intentionally blurred for anonymity.

The Danmaku-based and Synchronous (DS) feedback was used as a mode of peer feedback for sessions of L2 oral English conducted via live streaming platforms. The online streaming of L2 oral English courses and practices used VooV Meeting. Students are encourage to provide feedback for their peers through Danmaku, viewer-submitted subtitles that were synchronized to the video timeline [56,58]. See Fig 1 for a screenshot of online L2 oral English session with peer feedback displayed on screen as Danmaku.

#### Danmaku-based and asynchronous feedback

The Danmaku-based and Asynchronous (DA) feedback was the alternative to DS feedback. The variance between DA and DS was that the former was submitted as summative feedback during a discussion session at the end of the livestreaming (during instruction, Danmaku was deactivated).

#### Oral and synchronous feedback

The Oral and Synchronous (OS) feedback was the frequently used in L2 oral English sessions. In an oral English session adopting OS, the lectures encouraged students to observe the performance of peer students and provide immediate peer feedback regarding the performance of their peers’ L2 oral production. In most cases, the OS feedback were provided orally during the teaching and practicing sessions.

#### Written and asynchronous feedback

**Fig 2:**
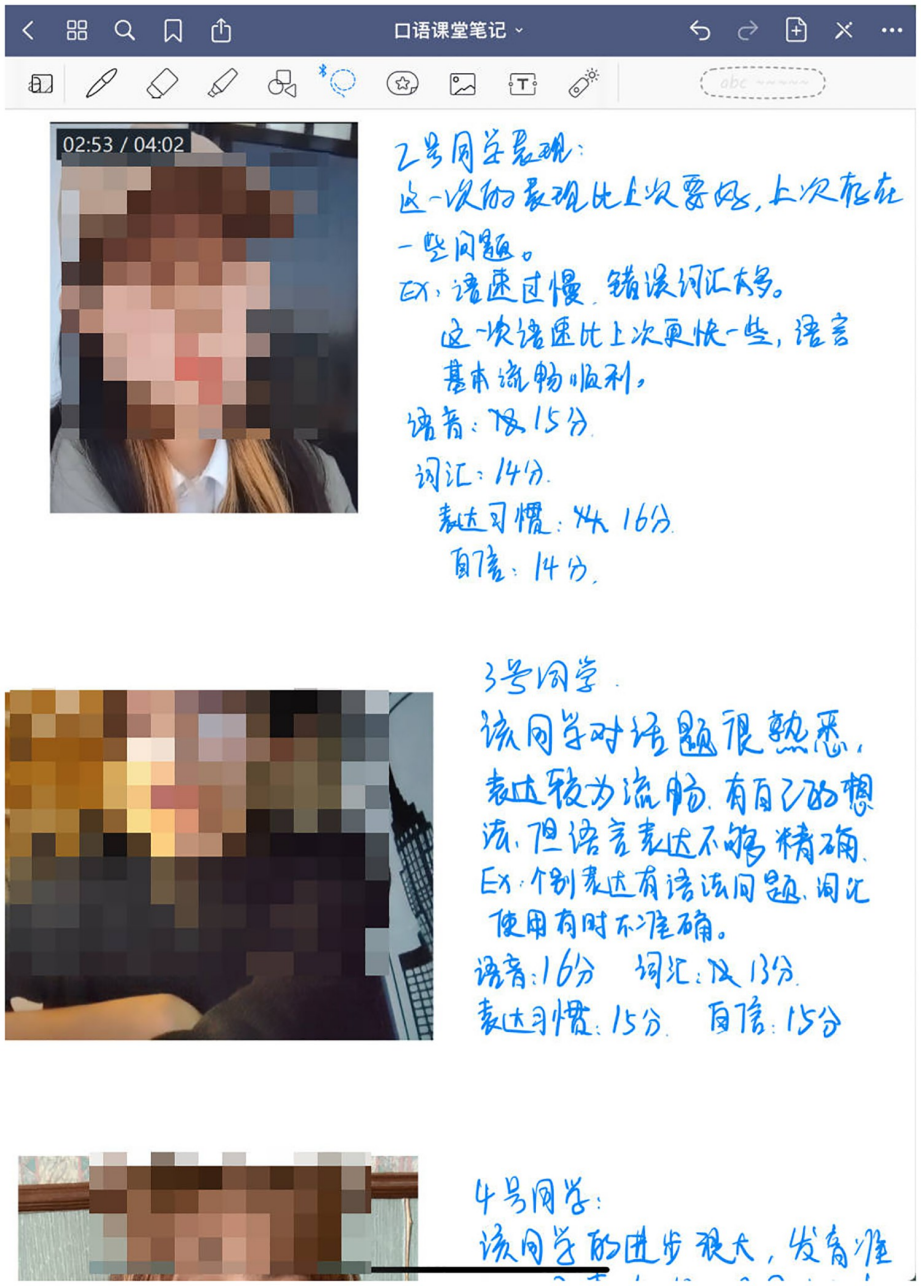
Students’ E-notes of written and asynchronous peer feedback. Note: Names and faces of students were intentionally blurred for anonymity.

The Written and Asynchronous (WA) feedback was the conventional mode of peer feedback used in learning English as a foreign language at the university. In a typical L2 oral English course, the lectures encouraged students to observe the performance of peer students and submit a post-session report in which peer feedback was provided. In most cases, the WA feedback were submitted or reviewed in paper-pen notes or electronic notes. See Fig 2 for a sample of students’ WA feedback in electronic notes.

### 2.4 Experimental design and procedures

**Fig 3:**
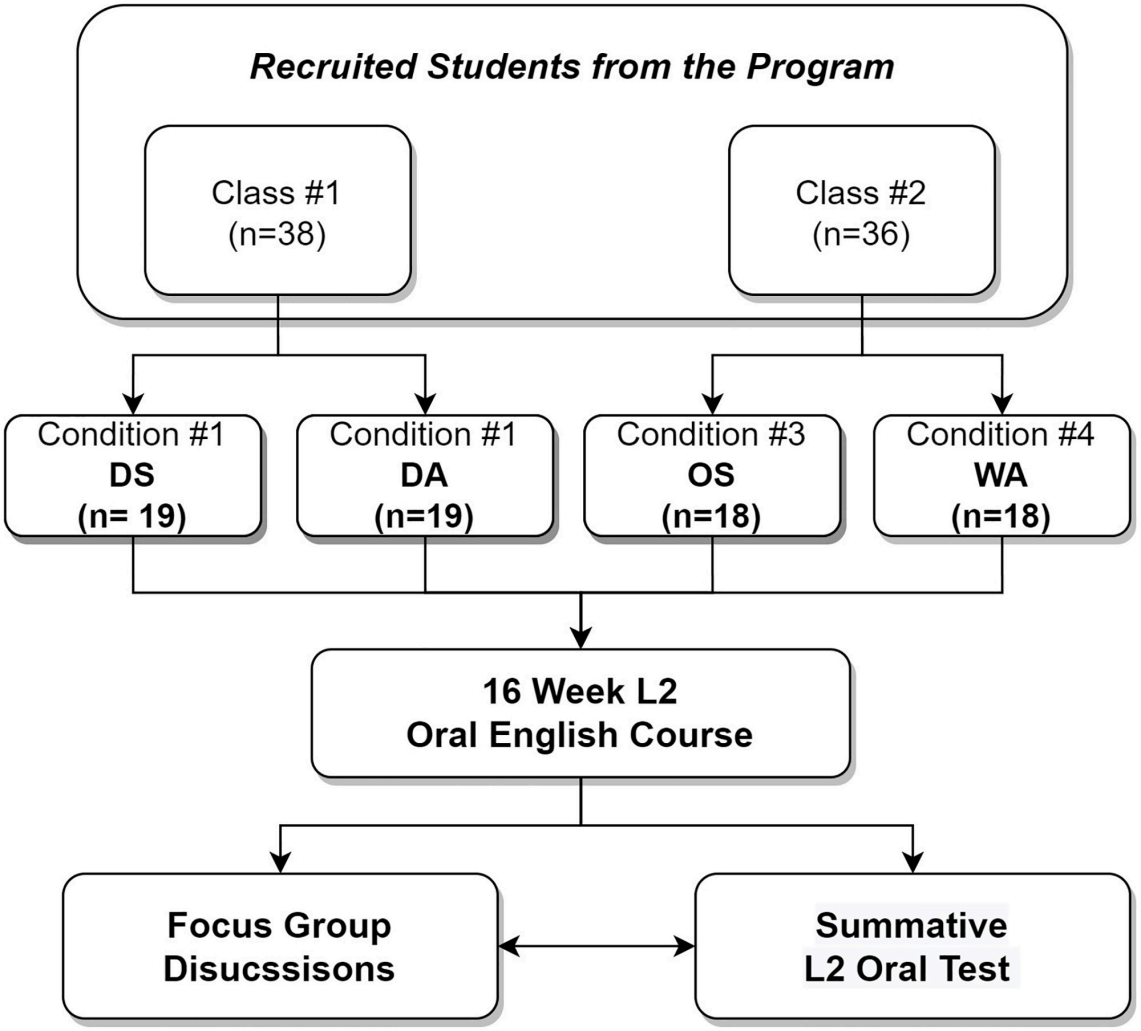
Experimental design of the present study. Notes: OS: Oral and synchronous feedback; WA: Written and asynchronous feedback; DS: Danmaku-based and synchronous feedback; DA: Danmaku-based and asynchronous feedback.

A between-subject factorial design was applied in the present study to investigate the impact of Danmaku-based and synchronous feedback on students’ L2 oral performance in a summative test [67]. Specifically, the 2x2 design encompassed four different conditions: OS, WA, DS and DA. In the experiment, students from class #1 were randomly assigned to the two condition groups using Danmaku-based feedback: DS group (n = 19) and DA group (n = 19); students from class #2 were randomly assigned to the two condition groups using non-Danmaku feedback: OS group (n = 18) and WA group (n = 18). The duration of the experiment was 16 weeks, the same as the regular one-semester L2 oral English course for all other learners in the program. In the concluding week, a summative test on students’ L2 oral performances were administered. Additionally, in week 8 and 16, participants were requested to join two sessions of focus group discussion about their perceptions and reflections towards the different modes of peer feedback. To attain validity in measurement, all other aspects pertaining to the educational process, e.g., syllabus, didactic materials, and out-of-class activities, remained invariant. Additionally, an 1.5-hour pre-experimental training was provided to participants of each group to improve their understanding of the present study and the mechanisms of peer feedback to be used in teaching. See Fig 3 for an illustration of the experimental design of the present study.

### 2.5 Data collection

The summative test encompassed four L2 oral tasks, e.g., self-introduction, Q&A, comment on a given paragraph, and impromptu speech. The test was designed by following the test specification of the nationally accredited College English Test-Spoken English Test (CET-SET) in China. The CET-SET is a nation-level test widely applied in China’s higher educational systems for the evaluation of student’s L2 oral proficiency. Its reliability and validity have been testified and reported in previous studies [68]. Students’ L2 oral performance were graded against a rating rubric adapted from the evaluation rubric for the oral tests developed by Wu et al. [69]. The rubric encompassed five dimensions of oral performance: fluency, pronunciation, grammar, vocabulary and content knowledge [69]. For each dimension, raters graded on a five-point rating scale. Consequently, the final grade of a test taker was the aggregate of the grades in each dimension for four tasks. Two lectures served as raters for the summative test. When agreement could not be met regarding a student’s grade, a joint discussion with an additional rater would be convened until consensus was reached. According to measurement of Cohen’s kappa (κ), the inter-rater reliability (κ = .82) in the grading was of an acceptable level.

The focus group discussion (FGD) sessions lasted for approximately 45–60 minutes per session. Students were assigned to groups of 5 members under the guidance of two moderators. Both moderators received training and were informed of the study’s purposes and objectives. During the FGD sessions, one of the moderator took control and leaded the discussion, while the other moderator took down brief notes of the discussion and provided assistance if needed. FGD protocol was developed and strictly adhered to during each session. All FGD sessions were audio recorded and transcribed verbatim. All materials generated from the FGD sessions were submitted to the researchers upon agreement in the member-checking process [70].

### 2.6 Data analysis

Given the 2x2 factorial design of the experiment, the researchers initially planned to run a two-way ANOVA to examine the effects of peer feedback modes on learners’ L2 oral performance. However, according to the results of the Shapiro-Wilk test, only the aggregated grade data (W = .978, p = .238) satisfied the assumption of normal distribution for parametric ANOVA. Following the suggestion from Conover and Iman’s [71] work, rank transformation was performed for all the non-normally distributed data before a parametric two-way ANOVA on the data ranks in R statistic software (version 4.2.2).

For qualitative data, a deductive thematic analysis in accordance with the six-step procedures postulated by Braun and Clarke [72] was followed. Two co-researchers recruited from the lecturers at the university joined the researchers for coding and theme identification. Following the recommendations from Braun and Clarke [72], the analysis procedures included: 1) familiarization with the data; 2) generating codes; 3) initial themes extraction; 4) theme reviewing; 5) defining and finetuning of the themes; and 6) report production. The coders and the researchers collaborated in the analysis process and tried to solve disputed opinions through inter-coder and team consensus [73].

To ensure the trustworthiness of the qualitative strand, we had taken several measures. First, the overall qualitative data analysis process was overseen by an expert panel whose members were the deans and deputy deans from the research sites. Second, we adhered to the procedures proposed by Nowell et al. [74]. With the specific strategies, e.g., researcher triangulation [75], peer debriefing [76], and thick description of the context [77], the trustworthiness of the findings from the qualitative strand could be enhanced.

### 2.7 Ethics

This study was approved by the Curriculum Development Committee and Ethics Committee of School of Foreign Languages, Xinyang Agriculture and Forestry University. Written informed consents were obtained from all participants of the study.

## 3. Results

### 3.1 Effects of modes of peer feedback on L2 oral performances

Upon the completion of grading of the summative test, we conducted descriptive analysis on the data to obtain a glimpse of the test results. See Table 2 for the descriptive analysis results of the grades.

**Table 2:** Descriptive statistics of the summative test of L2 oral performance.

	Group	Mean	SE	Median	SD	Minimum	Maximum
Total	WA	64.94	0.769	65.0	3.26	59	71
	OS	70.22	0.613	70.0	2.60	64	75
	DA	71.63	0.806	72	3.52	67	81
	DS	80.11	0.709	80	3.09	76	87
Fluency	WA	12.06	0.338	11.0	1.43	11	14
	OS	14.17	0.373	14.5	1.58	12	16
	DA	15.00	0.315	15	1.37	13	17
	DS	17.32	0.367	18	1.60	15	19
Pronunciation	WA	9.89	0.351	10.5	1.49	8	12
	OS	11.78	0.319	12.0	1.35	10	14
	DA	14.05	0.301	14	1.31	12	16
	DS	16.32	0.276	16	1.20	14	18
Grammar	WA	12.89	0.387	12.0	1.64	11	16
	OS	13.61	0.335	13.0	1.42	12	17
	DA	13.58	0.289	13	1.26	12	16
	DS	15.00	0.315	15	1.37	13	17
Vocabulary	WA	13.94	0.347	14.0	1.47	11	17
	OS	15.22	0.339	16.0	1.44	12	17
	DA	14.11	0.275	14	1.20	12	17
	DS	15.47	0.258	16	1.12	13	17
Content Knowledge	WA	16.17	0.326	17.0	1.38	13	17
	OS	15.44	0.390	15.0	1.65	13	18
	DA	14.89	0.366	15	1.59	11	17
	DS	16.00	0.286	16	1.25	14	18

**Fig 4:**
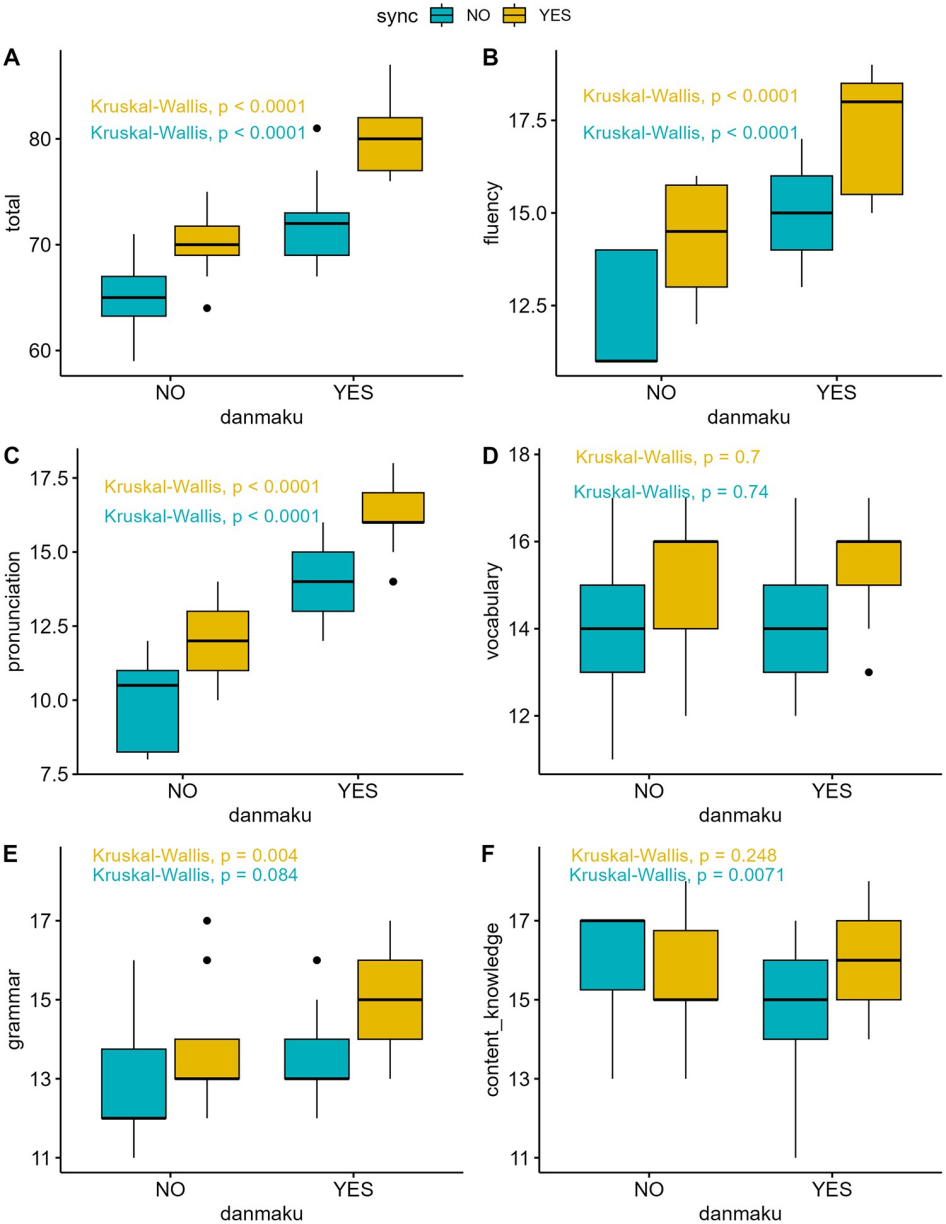
Boxplot of total and dimensional grades of the summative test.

According to the descriptive statistics, students from the WA group were believed to be low achievers among all participants (M = 64.94, SD = 3.26); students from the DS group were the high achievers (M = 80.11, SD = 3.09); students from OS group (M = 70.22, SD = 2.60) and DA group (M = 71.63, SD = 3.52) formed the middle achievers with similar summative grades. See Fig 4A for the boxplot of the total grade of the summative test. For the five dimensions within the L2 oral production abilities, the descriptive analysis manifested that.

Regarding the fluency dimension within the summative test results, students’ performances were in a similar vein as their total grades, with students in WA groups (M = 12.06, SD = 1.43) lagging behind OS group (M = 14.17, SD-1.58) and DA group (M = 15.00, SD = 1.37) and DS group (M = 17.32, SD = 1.60). See Fig 4B for the boxplot of the fluency grades.

In the measurement of students’ pronunciation abilities, students from the DS group were leaders with observable advantage (M = 16.32, SD = 1.20), followed by DA group (M = 14.05, SD = 1.31) and OS group (M = 11.78, SD = 1.34) and WA group (M = 9.89, SD = 1.49). See Fig 4C for the boxplot of the pronunciation grades.

Student’s mastery of grammar reflected in their oral production were basically on par, with OS group (M = 13.61, SD = 1.42) leading marginally WA group (M = 12.80, SD = 1.64) and DA group (M = 13.58, SD = 1.26), except for students from DS group (M = 15.00, SD = 1.37). See Fig 4D for the boxplot of the grammar grades.

For vocabulary abilities in the L2 oral production, synchronous groups outran their asynchronous counterparts, with DS group (M = 15.57, SD = 1.12) leading OS group (M = 15.22, SD = 1.44), DA group (M = 14.11, SD = 1.20) and WA group (M = 13.94, SD = 1.47). See Fig 4E for the boxplot of the vocabulary grades.

For the measurement of content knowledge, students from WA group (M = 16.17, SD = 1.38) were high achievers, followed by DS group (M = 16.00, SD = 1.25), OS group (M = 15.44, SD = 1.65) and DA group (M = 14.89, SD = 1.59). See Fig 4 for the boxplot of the content knowledge grades.

To understand the effects of various peer feedback modes on L2 oral performance, we used two-way ANOVA to compare the differences in grades among four groups. See Table 3 for the results.

**Table 3:** Two-way ANOVA results.

		Sum of Squares	Df	Mean Square	F	P
Total	danmaku	1269	1	1268.96	128.69	< .001***
	sync	874	1	873.96	88.63	< .001***
	danmaku * sync	47.2	1	47.2	4.79	0.032*
Fluency	danmaku	13121	1	13121	70.33	< .001***
	sync	6967	1	6967	37.34	< .001***
	danmaku * sync	0.04	1	0.04	0.084	0.73
Pronunciation	danmaku	21881	1	21881	216.442	< .001***
	sync	4344	1	4344	42.975	< .001***
	danmaku * sync	77	1	77	0.766	0.384
Vocabulary	danmaku	102	1	102	0.286	0.594
	sync	7261	1	7261	20.290	< .001***
	danmaku ideo * sync	26	1	26	0.074	0.787
Grammar	danmaku	3856	1	3856	11.007	0.001**
	sync	3782	1	3782	10.794	0.002**
	danmaku * sync	283	1	283	0.808	0.372
Content Knowledge	danmaku	546	1	546.4	1.329	0.253
	sync	32	1	32.4	0.079	0.8
	danmaku * sync	2945	1	2945.2	7.165	0.01**

Note:

* p < .05;

** p < .01;

*** p < .001.

The two-way ANOVA was performed to analyze the effects of channel (i.e., Danmaku-based or written/oral) and timing (i.e., synchronous or asynchronous) of peer feedback on students’ performance in L2 oral production.

**Fig 5:**
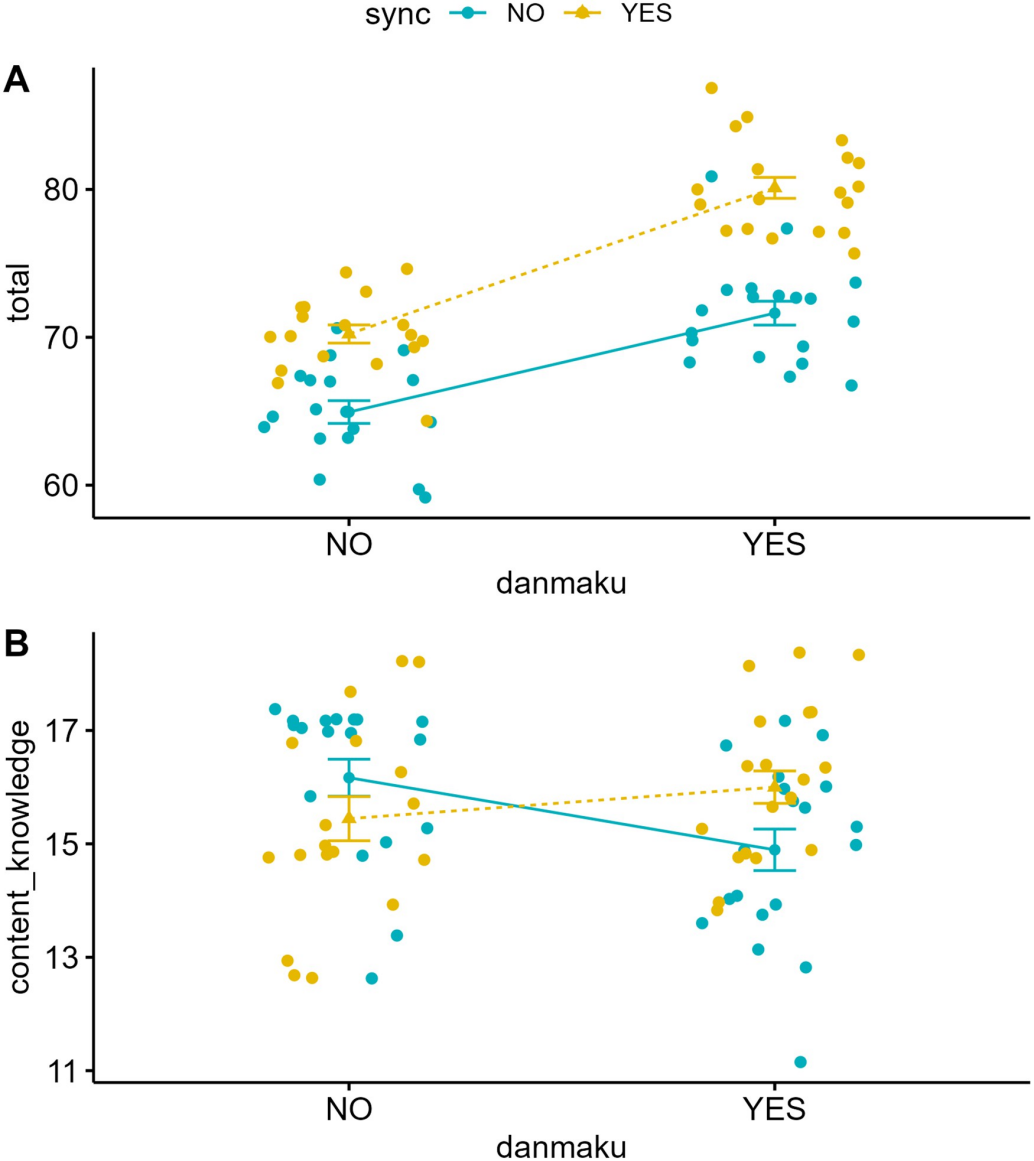
Interaction plots of total and content knowledge grades of the summative test.

The results from the two-way ANOVA revealed that channel (p < .001) and timing (p < .001) both had a statistically significant effect on students’ L2 oral production performances. Additionally, there was a statistically significant interaction between the effects of channel and timing of peer feedback (F(1, 70) = 4.79, p = .032). Since a statistically significant interaction between channel and timing of peer feedback was identified, a post-hoc test for pairwise comparison was conducted using Tukey’s Honestly-Significant-Difference (TukeyHSD) test. The Tukey post hoc test showed that the total grades of the students from the DS group were statistically significantly greater than those from other groups (p < .001). See Table 4 for the results of the post-hoc comparison on the effects of the interaction between channel and timing of peer feedback on L2 oral performance. The interaction between the two variables was shown in Fig 5A.

**Table 4:** Post-hoc comparisons on the effects of interaction between channel and timing of peer feedback on total L2 oral performance.

Comparison											95% CI	
danmaku	sync		danmaku	sync	Mean Difference	SE	df	t	p_tukey_	Cohen’s d	Lower	Upper
NO	NO	-	NO	YES	-5.28	1.05	70.0	-5.04	< .001	-1.681	-2.403	-0.958
		-	YES	NO	-6.69	1.03	70.0	-6.47	< .001	-2.130	-2.877	-1.382
		-	YES	YES	-15.16	1.03	70.0	-14.68	< .001	-4.828	-5.873	-3.783
	YES	-	YES	NO	-1.41	1.03	70.0	-1.36	0.526	0.449	-0.212	1.109
		-	YES	YES	-9.88	1.03	70.0	-9.57	< .001	-3.147	-3.991	-2.304
YES	NO	-	YES	YES	-8.47	1.02	70.0	-8.32	< .001	-2.698	-3.489	-1.907

For the effects on students’ L2 oral fluency, there was no statistically significant interaction between channel and timing of peer feedback (F(1, 70) = .084, p = .73). The simple main effects analysis showed that channel (p < .001) and timing (p < .001) both had a statistically significant effect on students’ L2 oral production fluency. Similarly, there was no statistically significant interaction between channel and timing of peer feedback in the measurement of pronunciation (F(1, 70) = .766, p = .384). The simple main effects analysis showed that channel (p < .001) and timing (p < .001) both had a statistically significant effect on students’ L2 pronunciation.

For the effects of the channel and timing of peer feedback on L2 vocabulary abilities reflected in the summative test, there was no statistically significant interaction between channel and timing of peer feedback (F(1,70) = .074, p = .787). The channel of peer feedback didn’t have a statistically significant effect on vocabulary abilities (p = .594), while the timing of peer feedback had a statistically significant effect (p < .001).

For the effects on students’ grammar abilities reflected in L2 oral production, there was no statistically significant interaction between channel and timing of peer feedback (F(1, 70) = .808, p = .372). The simple main effects analysis showed that channel (p < .001) and timing (p < .001) both had a statistically significant effect on students’ L2 grammar abilities.

In the measurement of content knowledge abilities in L2 oral production, neither channel (p = .253) nor timing (p = .8) of peer feedback had a statistically significant effect on such ability. However, the interaction between the channel and timing of peer feedback was statistically significant (F(1,70) = 7.165, p < .01). According to the results of a post-hoc comparison using TukeyHSD, the differences in grades among all four groups were statistically insignificant (p>.05). See Table 5 for the results of the post-hoc comparison. The interaction between the two variables was shown in Fig 5B. Combining the post-hoc comparison results and the interaction plot, the positive effects of Danmaku-based feedback on synchronous feedback and the negative effects on asynchronous feedback abilities could be identified though statistically insignificant among groups.

**Table 5:** Post-hoc comparisons on the effects of interaction between channel and timing of peer feedback on content knowledge in L2 oral performance.

Comparison											95% CI	
danmaku	sync		danmaku	sync	Mean Difference	SE	df	t	p_tukey_	Cohen’s d	Lower	Upper
NO	NO	-	NO	YES	0.722	0.492	70.0	1.467	0.463	0.489	-0.181	1.1588
		-	YES	NO	1.272	0.486	70.0	2.618	0.052	0.861	0.189	1.5330
		-	YES	YES	0.167	0.486	70.0	0.343	0.986	0.113	-0.543	0.7691
	YES	-	YES	NO	0.550	0.486	70.0	1.131	0.671	-0.372	-1.031	0.2869
		-	YES	YES	-0.556	0.486	70.0	-1.143	0.664	-0.376	-1.035	0.2830
YES	NO	-	YES	YES	-1.105	0.479	70.0	-2.306	0.106	-0.748	-1.408	-0.0890

### 3.2 Experiences and reflections from participants

**Fig 6:**
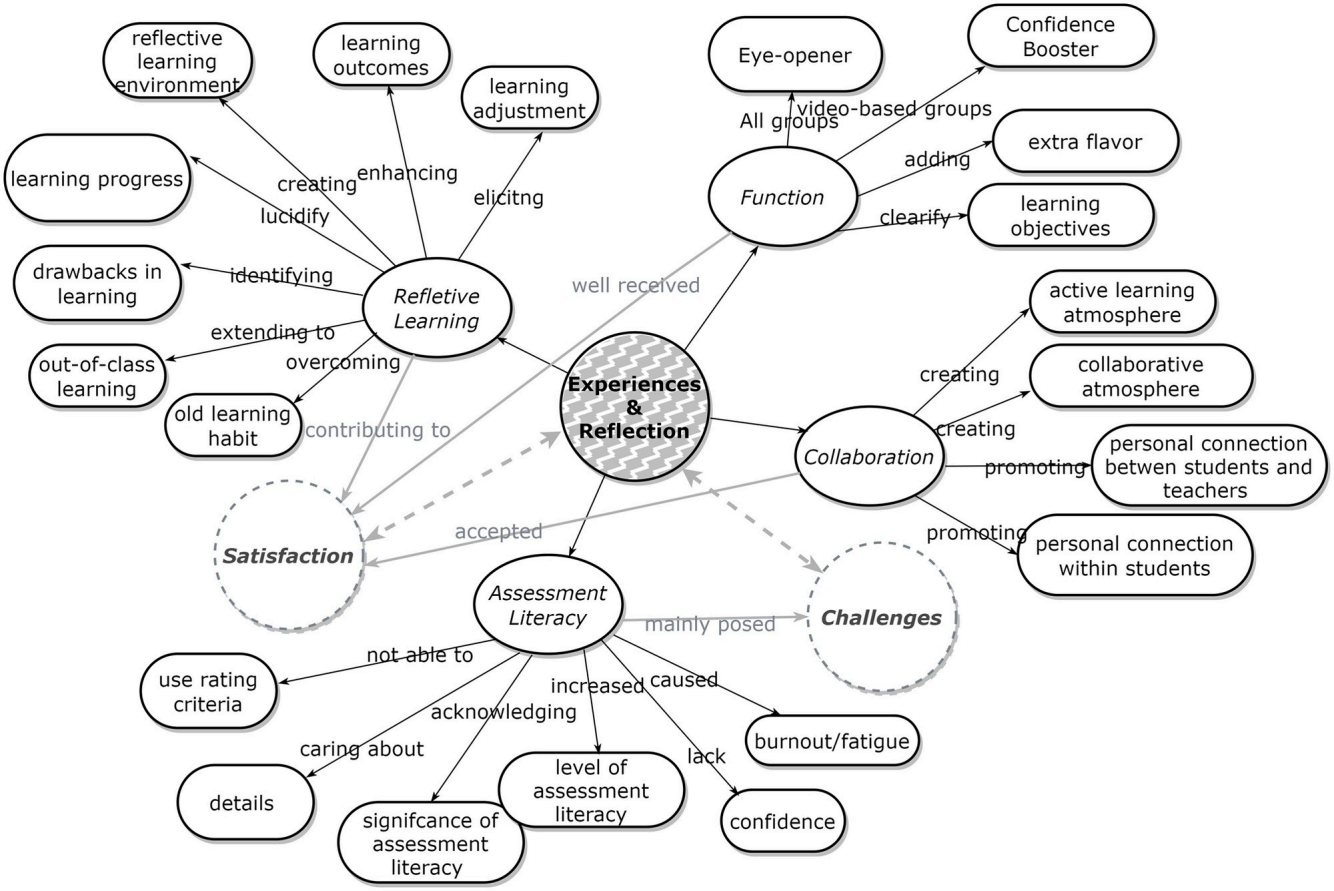
Thematic map of the qualitative findings.

In the qualitative strand of the present study, participants in the experiment joined the FGD in 5-member groups. Based on the synthesized results from the FGD, a series of themes regarding their perceptions towards the efficacy and experiences of peer feedback were identified. See Fig 6 for the thematic map of the qualitative findings of the present study.

#### 3.2.1 Reflective learning

When asked about the change experienced with adoption of peer feedback as a regular didactic component in L2 oral English classroom, most students reported that they were empowered by the provision of opportunity for a “reflective learning” environment. Reflective learning, as observed by the participants, referred to the learning experience in which they could assess their skills actively throughout the process.

To participants, one of the most significant efficacies of peer feedback was the chance for them to lucidly understand their current learning status and drawbacks in their learning strategies. As a student from the OS group claimed:

“[the most dramatic differences from other classes I experienced] is the ability to know my own issues in learning oral English. While in other classes, we could receive feedback from the lecturer, but the chances were just minimal, may be three or four times a semester, and it’s not enough at all”

(FGD-OS#2-#03).

Regarding the reflective activities, students stressed that the impact was not only limited to classroom sessions. To them, the peer feedback received during classroom sessions normally continued to influence their follow-up learning activities, e.g., self-directed learning after class, learning group activities and even self-reflection on their learning progressions. In their words, the feedback helped them to be engaged in a “continuous reflective process” to see themselves better.

Additionally, students argued that the feedback received from their peers could be beneficial for their adjustment of learning strategies or plans. To novice learners in college courses, the incompatibility between their inherited learning habit from middle school with the reality and requirement of the curriculum of English majors posed major challenges. As reflected by a participant from the DA group:

“[in the middle school] we didn’t have any listening and oral English drills. I am just a newbie facing the new oral English courses. I have literally experienced the shift from a not well-prepared status to a more prepared learner. I have to thank my peers, especially my fellow students in the same group, who have helped me a lot”

(FGD-DA#1-#04).

Among all participants, students from the DA group and DS group generally expressed their satisfaction on the Danmaku-based approaches of peer feedback. According to their discussions during the FGD sessions, the strength of Danmaku-based peer feedback was the possibility to acquire a clearer impression of the performance of the peer learners. Exclusively for participants of the DS group, they were supportive to the peer feedback mode and wished the lecturers to adopt such peer feedback in other courses. As a student from the DS group argued:

“I know much about Danmaku, I use it daily on video-sharing platforms. The chance to use it in learning English shocked, impressed and amazed me. I think instant feedback from your peers is so interesting, and [most importantly], you can get back to the moment anytime afterwards, still being amazed as before”

(FGD-DS#2-#05).

#### 3.2.2 “Eye-opener” and “Confidence Booster”

When asked to use a nominal phrase to denote the function of the peer feedback, different voiced were heard from responses between different groups. Participants from the non-Danmaku groups (i.e., the WA and OS groups) were in agreement for words such as “eye-opener” or “extra flavor” for the description of the role played by the peer feedback in L2 oral English learning. Conversely, participants from the Danmaku-based groups moved one step further. To them, the functions of peer feedback were not only a bringer of new perspectives but also a booster of their confidence as English majors. Such insight could be observed from a FGD discussion in a mixed group, in which members from four groups were included:

“Student #03: I agree that the peer feedback serves like a microscope with which we can know more about ourselves. What’s more, it let us know more about what we can learn, what can we do and how to do if we are not so good. It broadens our horizon.

Student #05: Since we are receiving feedback through the Danmaku which is synchronized with the videos, I think it could increase our confidence. I am afraid of being criticized by others, but when the criticism works, you will like it and want more of it.

Student #03: You will always look back at your videos with Danmaku?

Student #05: Not only that, but we also wish to have more chances to speak in front of the class as well. When you can see your progress, you will be glad.” (FGD-Mixed Group #2-Session#2).

The incorporation of peer feedback granted students more chances to have a clearer understanding of the learning objectives of the course. In classroom activities, students not only learned from the lecturer and the didactic materials provided, but they could learn from their peers. Especially for the learning of oral English, in which students showed relatively larger personal differences in their capabilities, the “peer feedback—peer learning—peer motivation” chain augmented the learning experiences. As supported by two participants:

“I enjoy learning form my fellow students. It is just easier, more casual and more fun. I think the process [of peer learning] improved the original instruction-dominated classroom environment”

(FGD-DS#1-#03).

“Besides the ability for learning improvement, I am mentally more powerful than I used to be. I am always encouraged and praised by other students. I have to confess that I love it”

(FGD-WA#2-#04).

Most importantly, the feedback was both effective and easier to be accepted by students. For novice college learners, peer feedback, even with negative remarks and criticisms, were easier to be accepted. Students were mentally unprepared for lecture’s feedback and remarks, as the judgment from lectures were high-stakes ones. As a student said, the peer feedback was “lighter, smoother and less harsh”. In turn, peer feedback could be deemed as a balance between effectiveness and gentleness, which eventually contributed to their increasement in confidence. As a student asserted:

“You can’t gain confidence from not doing anything. But in reality, sometimes you get corrupted faith in yourself after your initial attempts. So far, I think peer feedback could rescue us from the plighted situation”

(FGD-DA#2-#01).

#### 3.2.3 Collaboration in learning

Satisfaction and acceptance towards the collaborative atmosphere were shared by most of the participants. To the students, collaboration with their peer learners were manifold. First, through peer feedback, a connection between the oral presenter and the feedback provider was established. In a conventional classroom, the direct interaction between students were limited and intentionally underplayed for the alleged negative impact on the flow of instruction and classroom activities. According to the participants, peer feedback not only enabled them to spot flaws in the oral production from peer learners, but also granted them chances to seek learners with similar habit or learning abilities. Student emphasized that the bond between co-learners was enhanced and proved beneficial for their learning. Second, in a broader context, the learning of L2 oral English among learners shifted towards a collaboration-based mode. The relatively macroscopic change taken place in the L2 oral English classroom was favored by most students. In a conventional classroom of oral English, the interaction between lectures and students and within students were planned and prescribed in the syllabus. With the ambience of collaborative learning mediated by the inclusion of peer feedback in pedagogy, collaborative learning between students were more frequent and effective. As affirmed by a student:

“When we were first encouraged to contribute to the peer feedback, we were shy and slow. Now we are different. Everyone seems to be enjoying it and learning with it as a habit. Even in out-of-class activities, we are willing to be proactive in feedback”

(FGD-DA#2-#05).

#### 3.2.5 Assessment literacy

Pertinent to the challenges encountered during peer feedback, a unison was heard among the participants that they were less confident in their abilities for objectively and comprehensively provide effective feedback for peer learners. The self-perceived lack of assessment literacy was primary reflected in the following aspects:

First, students were not apt to use rating criteria objectively. Though a pre-experimental training session on fundamental knowledge in assessment and rating were provided to all participants, their ability to wield the rating instrument remained limited. For participants, this could be attributed to their lack of experience and training in assessment. In their words, they are “more experienced to be assessed and judged” instead of the way around. As reported by a student:

“I am regretful for my lack of ability to provide objective feedback. The descriptions in the rubric just didn’t work for me. I am hesitant to make judgement, especially in a timely manner. In server times, the moment I regretted for my decision the moment I clicked the submit button”

(FGD-DS#1-#04).

Second, student tended to be focused on the details in oral production. A common experience shared by participants in the FGD sessions was that they lacked a sense to capture the “broader picture”. Instead, students confessed that they were more interested and focused on the minute details in oral production, e.g., the choice of a certain word, a singular pronunciation imperfection, or even a habitual paralinguistic behavior. However, an opposing sound from another cohort within the participants was heard that details should be paid special attention. The disagreement reflected the preference and rejection for a holistic perspective in peer feedback. As a student argued in a FGD session:

“I used to be keen on the details, spending several seconds pondering over the ‘errors’ I have sensed. But after a semester of providing and receiving feedback from the learning community, I am changing my mindset gradually. I think the overall quality prevails”

(FGD-WA#2-#02).

Third, students were not confident in the quality of their feedback provided to peer learners. This would be understandable as they were novice college students themselves. Participants responded that their feedback risks of bearing minimal value. Some students believed that the quality of peer assessment would gradually improve alongside with their accumulation of experiences and competencies. From another angle, students also believed that amateurishness and casualness were the innate features of peer feedback. As reported by a FGD member:

“I think it would be hard for us to give very professional feedback. We are not professionals or lecturers. But I think this happens to be the beauty of peer feedback, isn’t it?”

(FGD-DA#1-#04).

Fourth, a sense of “burnout” or fatigues was reported by participants due to the lack of experience and proficiency in providing peer feedback. According to the respondents, the tension experienced in providing feedback was similar to that of performing L2 oral tasks. Students were in agreement in contributing the reason to lack of assessment literacy and experiences thereof. As a student said, “making mistakes in providing feedback was even worse than making mistakes in doing the task” himself.

Nevertheless, students were affirmative that their level of assessment literacy was significant improved through their involvement in peer feedback and relevant formative assessment activities in classroom. An agreement regarding the position of peer feedback in language learning was reached. As a student argued:

“Seeing other student presenting their learning outcomes is also learning to me. I think I am a better feedback provider than I used to be. I am really happy to be able to learn while watching and thinking about my own abilities if I were assigned the task”

(FGD-WA#2-#05).

## 4. Discussion

The research set out to examine the impacts of the interplay between different channel and timing of peer feedback on learners’ L2 oral performance. The results revealed that Danmaku-based and synchronous feedback was effective in enhancing overall learning outcomes but had mixed effects on the subdomains of L2 oral competence. Furthermore, participating students expressed general satisfaction towards the incorporation of technology-mediated peer feedback in L2 classrooms.

### 4.1 Potential of Danmaku-based and synchronous peer feedback in L2 oral pedagogy

The statistical analysis of the study revealed that different modes of peer feedback would have variegated effects on students’ performance in L2 oral English summative test. In the field of L2 oral production, the attempts to incorporate peer feedback received similar findings. For example, in a study on impact of feedback on Bahraini university L2 learners’ oral presentation skills, feedback from both teachers and peers were believed to be positively effective [78]. In a similar fashion, the effects of peer supported feedback on learners’ French oral proficiency was asserted [27]. The empirical findings from the study contributed to expand our knowledge in the niche. Furthermore, the impact of technology-mediated peer feedback strategies (i.e., posting instant feedback via Danmaku, using video-recordings as references for feedback, submitting feedback in the form of electronic notes, etc.) proved both effective and favorable for participating students. The results have testified the claims that technology-mediated peer feedback would contribute to the enhancement of feedback literacy and uptake from Wood’s Work [79]. The findings from the present study manifested that the utilization of Danmaku-based peer feedback could positively impact on students’ abilities for L2 oral production.

From a theoretical perspective, the results from the study could be further explained as the effects of technology-enhance learning strategies on the socio-affective and relational aspects of feedback. Aligning the findings to the socio-constructivist learning theories, the purpose of using peer feedback in L2 pedagogy could be repositioned as creating a new communication channel for peer learners [80]. As argued by Rambe [81], the cognitive scaffolding ushered in by the introduction of technology-mediated peer feedback promoted learners’ affective attitudes and engagement in learning. Sharing the views of Emmerson [82] that the combination of the advancement in both technology and sociocultural learning environment would further improve the effects of language learning, the findings of the study contributed to expand our empirical knowledge in this field.

### 4.2 Mixed effects of feedback modes on learning outcomes

The most interesting finding from the first strand of the study would be the varied effects of the peer feedback on the subdomains of L2 oral performance. Specifically, the effects of multiple modes of peer feedback on students’ vocabulary and content knowledge abilities were minimal, compared to other three dimensions. The dramatic variance among the effects between different L2 oral sub-competencies were the unexpected findings of the present study. The underlying reason for the mixed findings in vocabulary abilities could be attributed to the fact that students learned new vocabulary incidentally in the study. In a game-enhanced learning environment, the researchers argued that incidental acquisition of vocabulary knowledge was always a relatively slower process [83]. Empirical findings from a language learning environment adopting video-dubbing as learning strategies shared such views [84]. The limited effects of the feedback modes on content knowledge acquisition could be partially explained in a similar fashion. Furthermore, the unexpected results could be attributed to the design of the study. Instead of adopting a holistic approach to examine the effects of peer feedback on students’ performance, the study adopted a dimension-specific approach on top of a holistic analysis. In previous studies, the effect of feedback on L2 abilities were limited to the holistic performance or a specific domain of L2 abilities, e.g., improving oral fluence of English majors [85], using technology-enhanced tools to improve overall L2 production proficiency [86], utilizing multiple task types to increase L2 oral production competence [87]. The finding from the present study were basically in line with the outcomes from previous literature.

### 4.3 Students’ perceptions towards the Danmaku-based and synchronous peer feedback

In the present study, the implementation of regular peer feedback was well received by participants. For decades, successful cases have been documented to utilize feedback (e.g., peer feedback, teachers feedback and feedback based on self-assessment) for enhancement in both learning progression and motivation [23,28–30]. The findings of present study were in agreement with those from previous literatures. For example, in a blended learning environment, the adoption of formative feedback was asserted to be impactful on student’s engagement [88]. In the present study, students reported that the inclusion of peer feedback not only resulted in augmented learning experiences but also improved confidence and interest. By the same token, the findings that students appreciated the shift from keenness on grades to classroom feedback from a study on medical students was shared by the synthesized reflections of participants in the present study [89]. However, contradictory findings were observed against the claims from previous literature. For example, in a K-12 study on the effects of positive feedback on learning motivation, the researchers argued that positive feedback could not trigger educational or mental changes for an intrinsically motivating task [90]. In the present study, students reported that they proactively embraced the shift from a conventional learning environment to a collaborative one including frequent peer feedback. The difference in the findings between the present study and previous case could be attributed to the disparities in mental stability and receptiveness between college students and pupils in early adolescence [91].

### 4.4 Implication for research and pedagogy

The findings from the present study could trigger application of danmuku-based and synchronous feedback in L2 oral English education both theoretically and practically.

First, the present study would enlarge our conceptual and theoretical understanding of technology-enhanced peer feedback. In recent years, major advancements in educational technologies have been attained through the relentless efforts of educators and researchers [92]. In the field of reflective learning, initial attempts have been made to improve the efficacy and experience of education [93,94]. However, in the education of L2 oral abilities, the role played by technology-enhanced learning strategies or pedagogical instrument remain trivial. The present research indicated that the incorporation of technology-enhanced peer feedback would positively impact students’ learning. Most importantly, the present study tried to examine the effects of modern learning techniques on the internal dimensions within a certain language competency. The empirical findings could be used to form the in-depth conceptualization and contextualization of both peer feedback and the pedagogy of L2 oral abilities.

Second, the peer feedback design introduced in the study could be applied in authentic L2 education settings. In sharp comparison to the wide application of technology-enhanced peer feedback or peer assessment in other educational domains, notably the pedagogy of L2 writing, the application of technology-enhanced reflective learning strategies in L2 oral education was rather limited. In the present study, we have examined the differences between four modes of peer feedback on students’ learning outcomes and their perception thereof. From the four modes of peer feedback, Danmaku-based and synchronous feedback was the most significantly impactful and well-received by participants. According to previous studies, the power of technology-enhanced feedback has been testified in a series of different settings, e.g., the training of pre-service teachers [45], training of elite athletes [46], improvement of medical techniques [47], etc. However, it should be warned against that the implementation of peer feedback was not always smooth and well-acclaimed. In the study using feedback on student assessment, the researchers identified a series of potential weaknesses including students’ mental wellbeing and their abilities to wield the power of the feedback [95]. According to the reflection of participants in the present study, the utilization would cause similar mental burden and fatigue. Consequently, in authentic pedagogical settings, lecturers and program stakeholders should pay more attention to students’ psychological wellbeing and mental status. In the present study, we have found that the effects of peer feedback on different sub-competencies or dimension of a learning objective would vary dramatically. Consequently, a well-designed peer feedback should be selective in its scope and easy-to-implement.

### 4.5 Limitations and future directions

The present research was confided by several limitations, e.g., the relative short duration of experiment, and the small number of samples involved in the study.

First, the duration for the experiment was limited. Though at the university, a majority of courses were taught in 32 teaching hours, the training of L2 oral competence was estimated to take a much longer period of time. The design of the experiment allowed us to observe and examine the effects of different modes of peer feedback on educational outcomes. But given the limited duration, the learning outcomes and relevant implications to research and pedagogy risked potential bias. To curb the possible threats posed to the credibility of the study, we added extra-curricular learning activities alongside classroom instructions to increase time and opportunities for students to learn and practice L2 oral English.

Second, the number of subjects recruited for the experiments were limited. Given the limited resources and abilities to conduct empirical research at the university, a larger sample would new challenges to teaching and learning. Additionally, other language majors (i.e., translation and interpreter trainees at the same university) were excluded from the population. By recruiting only Business English majors, we could control the possible bias caused by the variances between the specification of curricula for different programs. However, the findings from the experiment risked of limited value for the reference of a different educational setting.

In successive studies, these limitations should be considered and addressed for more insightful findings regarding application of peer feedback in L2 oral English education. Additionally, compared to the fruitful findings of using technology-enhanced assessment or feedback strategies in other domain of language learning, the teaching of L2 oral abilities was in dire need for pedagogical innovations. From another angle, the assessment and evaluation of L2 oral abilities needed a more comprehensive and dynamic instrument other than the summative test used in the present study. In a nutshell, development of measurement instrument and technology-enhance assessment/feedback strategies should be encouraged and attempted for follow-up research.

## 5. Conclusions

The present study set out to examine the effects of different modes of peer feedback on students’ learning outcomes in L2 oral production after a 16-week experiment. Among all implemented modes of peer feedback, Danmaku-based and synchronous peer feedback outran its counterparts with the most significant impact in enhancing student’s level of L2 oral abilities. The study would contribute to the expansion of our conceptual knowledge and practical experience in peer feedback and L2 education. In follow-up studies, researchers could delve deeper into the measurement of oral abilities and state-of-the-art educational feedback and assessment strategies oriented for learning.

## References

[1] Adie L, Addison B, Lingard B. Assessment and learning: an in-depth analysis of change in one school’s assessment culture. Oxford Review of Education. 2021;47: 404–422. doi: 10.1080/03054985.2020.1850436

[2] Gibbs G. Why assessment is changing. Innovative Assessment in Higher Education. Routledge; 2006. pp. 31–42

[3] Morris R, Perry T, Wardle L. Formative assessment and feedback for learning in higher education: A systematic review. Review of Education. 2021;9: e3292. doi: 10.1002/rev3.3292

[4] Hattie J, Timperley H. The Power of Feedback. Review of Educational Research. 2007;77: 81–112. doi: 10.3102/003465430298487

[5] Zhang L, Zheng Y. Feedback as an assessment for learning tool: How useful can it be? Assessment & Evaluation in Higher Education. 2018;43: 1120–1132. doi: 10.1080/02602938.2018.1434481

[6] Wiliam D. Feedback: At the heart of—but definitely not all of—formative assessment. The Cambridge handbook of instructional feedback. New York, NY, US: Cambridge University Press; 2018. pp. 3–28.

[7] Kluger AN, DeNisi A. The effects of feedback interventions on performance: A historical review, a meta-analysis, and a preliminary feedback intervention theory. Psychological Bulletin. 1996;119: 254–284.

[8] Lui AM, Andrade HL. The Next Black Box of Formative Assessment: A Model of the Internal Mechanisms of Feedback Processing. Frontiers in Education. 2022;7. Available: https://www.frontiersin.org/articles/10.3389/feduc.2022.751548

[9] Winstone NE, Carless D. Designing effective feedback processes in higher education: a learning-focused approach. London; New York: Routledge, Taylor and Francis Group; 2020.

[10] Wisniewski B, Zierer K, Hattie J. The Power of Feedback Revisited: A Meta-Analysis of Educational Feedback Research. Frontiers in Psychology. 2020;10. doi: 10.3389/fpsyg.2019.03087 32038429 PMC6987456

[11] Ha XV, Nguyen LT, Hung BP. Oral corrective feedback in English as a foreign language classrooms: A teaching and learning perspective. Heliyon. 2021;7: e07550. doi: 10.1016/j.heliyon.2021.e07550 34337178 PMC8318991

[12] Yu S, Zhang Y, Zheng Y, Lin Z. Written Corrective Feedback Strategies in English-Chinese Translation Classrooms. Asia-Pacific Edu Res. 2020;29: 101–111. doi: 10.1007/s40299-019-00456-2

[13] Sailer M, Bauer E, Hofmann R, Kiesewetter J, Glas J, Gurevych I, et al. Adaptive feedback from artificial neural networks facilitates pre-service teachers’ diagnostic reasoning in simulation-based learning. Learning and Instruction. 2023;83: 101620. doi: 10.1016/j.learninstruc.2022.101620

[14] Cavalcanti AP, Barbosa A, Carvalho R, Freitas F, Tsai Y-S, Gašević D, et al. Automatic feedback in online learning environments: A systematic literature review. Computers and Education: Artificial Intelligence. 2021;2: 100027. doi: 10.1016/j.caeai.2021.100027

[15] Deeley SJ. Using technology to facilitate effective assessment for learning and feedback in higher education. Assessment & Evaluation in Higher Education. 2018;43: 439–448. doi: 10.1080/02602938.2017.1356906

[16] Andersson C, Palm T. Characteristics of improved formative assessment practice. Education Inquiry. 2017;8: 104–122. doi: 10.1080/20004508.2016.1275185

[17] Black P, Wiliam D. Developing the theory of formative assessment. Educ Asse Eval Acc. 2009;21: 5. doi: 10.1007/s11092-008-9068-5

[18] Hattie J. Visible learning: a synthesis of over 800 meta-analyses relating to achievement. London; New York: Routledge; 2009.

[19] Hattie J, Zierer K. Visible Learning Insights. 1st ed. Abingdon, Oxon; New York, NY: Routledge, [2019]: Routledge; 2019.

[20] Shang H-F. An exploration of asynchronous and synchronous feedback modes in EFL writing. J Comput High Educ. 2017;29: 496–513. doi: 10.1007/s12528-017-9154-0

[21] Shintani N, Aubrey S. The Effectiveness of Synchronous and Asynchronous Written Corrective Feedback on Grammatical Accuracy in a Computer-Mediated Environment. The Modern Language Journal. 2016;100: 296–319. doi: 10.1111/modl.12317

[22] Gaynor JW. Peer review in the classroom: student perceptions, peer feedback quality and the role of assessment. Assessment & Evaluation in Higher Education. 2020;45: 758–775. doi: 10.1080/02602938.2019.1697424

[23] Huisman B, Saab N, van den Broek P, van Driel J. The impact of formative peer feedback on higher education students’ academic writing: a Meta-Analysis. Assessment & Evaluation in Higher Education. 2019;44: 863–880. doi: 10.1080/02602938.2018.1545896

[24] Nicol D. Assessment for learner self‐regulation: enhancing achievement in the first year using learning technologies. Assessment & Evaluation in Higher Education. 2009;34: 335–352. doi: 10.1080/02602930802255139

[25] Holewik K. Peer Feedback and Reflective Practice in Public Service Interpreter Training. Theory and Practice of Second Language Acquisition. 2020;6: 133–159.

[26] Domínguez Araújo L. Feedback in conference interpreter education: Perspectives of trainers and trainees. Interpreting. 2019;21: 135–150. doi: 10.1075/intp.00023.dom

[27] Bedford S, Bissoonauth A, James K, Stace R. Developing a peer supported feedback model that enhances oral proficiency in French. Journal of University Teaching & Learning Practice. 2020;17. doi: 10.53761/1.17.5.13

[28] Irons A, Elkington S. Enhancing Learning through Formative Assessment and Feedback. 2nd ed. London: Routledge; 2021.

[29] Leighton JP. Students’ Interpretation of Formative Assessment Feedback: Three Claims for Why We Know So Little About Something So Important. J Educ Meas. 2019;56: 793–814. doi: 10.1111/jedm.12237

[30] Zhan Y, Wan ZH, Sun D. Online formative peer feedback in Chinese contexts at the tertiary Level: A critical review on its design, impacts and influencing factors. Computers & Education. 2022;176: 104341. doi: 10.1016/j.compedu.2021.104341

[31] Zhang J, Kuusisto E, Nokelainen P, Tirri K. Peer Feedback Reflects the Mindset and Academic Motivation of Learners. Frontiers in Psychology. 2020;11. doi: 10.3389/fpsyg.2020.01701 32765378 PMC7378527

[32] Hsia L-H, Huang I, Hwang G-J. Effects of different online peer-feedback approaches on students’ performance skills, motivation and self-efficacy in a dance course. Computers & Education. 2016;96: 55–71. doi: 10.1016/j.compedu.2016.02.004

[33] Zhang H, Song W, Shen S, Huang R. The Effects of Blog-Mediated Peer Feedback on Learners’ Motivation, Collaboration, and Course Satisfaction in a Second Language Writing Course. Australasian Journal of Educational Technology. 2014;30: 670–685. doi: 10.14742/ajet.860

[34] Fidan M, Gencel N. Supporting the Instructional Videos With Chatbot and Peer Feedback Mechanisms in Online Learning: The Effects on Learning Performance and Intrinsic Motivation. Journal of Educational Computing Research. 2022;60: 1716–1741. doi: 10.1177/07356331221077901

[35] Carless D, Boud D. The development of student feedback literacy: enabling uptake of feedback. Assessment & Evaluation in Higher Education. 2018;43: 1315–1325. doi: 10.1080/02602938.2018.1463354

[36] Zhu X, Evans C. Enhancing the development and understanding of assessment literacy in higher education. European Journal of Higher Education. 2022; 1–21. doi: 10.1080/21568235.2022.2118149

[37] Davari Torshizi M, Bahraman M. I explain, therefore I learn: Improving students’ assessment literacy and deep learning by teaching. Studies in Educational Evaluation. 2019;61: 66–73. doi: 10.1016/j.stueduc.2019.03.002

[38] Han Y, Xu Y. The development of student feedback literacy: the influences of teacher feedback on peer feedback. Assessment & Evaluation in Higher Education. 2020;45: 680–696. doi: 10.1080/02602938.2019.1689545

[39] Omar D, Shahrill M, Sajali M. The Use of Peer Assessment to Improve Students’ Learning of Geometry. European Journal of Social Science Education and Research. 2018;5: 187–206. doi: 10.2478/ejser-2018-0047

[40] Zhu Q, To J. Proactive receiver roles in peer feedback dialogue: Facilitating receivers’ self-regulation and co-regulating providers’ learning. Assessment & Evaluation in Higher Education. 2022;47: 1200–1212. doi: 10.1080/02602938.2021.2017403

[41] Kaya F, Yaprak Z. Exploring the Role of Training in Promoting Students’ Peer-Feedback Including Critical Peer-Feedback. Journal of Educational Research and Practice. 2020;10. doi: 10.5590/JERAP.2020.10.1.24

[42] Zong Z, Schunn CD, Wang Y. Learning to improve the quality peer feedback through experience with peer feedback. Assessment & Evaluation in Higher Education. 2021;46: 973–992. doi: 10.1080/02602938.2020.1833179

[43] Camarata T, Slieman TA. Improving Student Feedback Quality: A Simple Model Using Peer Review and Feedback Rubrics. Journal of Medical Education and Curricular Development. 2020;7: 2382120520936604. doi: 10.1177/2382120520936604 33029557 PMC7522828

[44] Darvishi A, Khosravi H, Abdi S, Sadiq S, Gašević D. Incorporating Training, Self-monitoring and AI-Assistance to Improve Peer Feedback Quality. Proceedings of the Ninth ACM Conference on Learning @ Scale. New York, NY, USA: Association for Computing Machinery; 2022. pp. 35–47.

[45] Prilop CN, Weber KE, Kleinknecht M. Effects of digital video-based feedback environments on pre-service teachers’ feedback competence. Computers in Human Behavior. 2020;102: 120–131. doi: 10.1016/j.chb.2019.08.011

[46] Nelson LJ, Potrac P, Groom R. Receiving video-based feedback in elite ice-hockey: a player’s perspective. Sport, Education and Society. 2014;19: 19–40. doi: 10.1080/13573322.2011.613925

[47] Schlick CJR, Bilimoria KY, Stulberg JJ. Video-Based Feedback for the Improvement of Surgical Technique: A Platform for Remote Review and Improvement of Surgical Technique. JAMA Surgery. 2020;155: 1078–1079. doi: 10.1001/jamasurg.2020.3286 32902619

[48] Dohms MC, Collares CF, Tibério IC. Video-based feedback using real consultations for a formative assessment in communication skills. BMC Medical Education. 2020;20: 57. doi: 10.1186/s12909-020-1955-6 32093719 PMC7041283

[49] Hung S-TA. Enhancing feedback provision through multimodal video technology. Computers & Education. 2016;98: 90–101. doi: 10.1016/j.compedu.2016.03.009

[50] Link S, Mehrzad M, Rahimi M. Impact of automated writing evaluation on teacher feedback, student revision, and writing improvement. Computer Assisted Language Learning. 2022;35: 605–634. doi: 10.1080/09588221.2020.1743323

[51] Fangohr H, O’Brien N, Hovorka O, Kluyver T, Hale N, Prabhakar A, et al. Automatic Feedback Provision in Teaching Computational Science. In: Krzhizhanovskaya VV, Závodszky G, Lees MH, Dongarra JJ, Sloot PMA, Brissos S, et al., editors. Computational Science—ICCS 2020. Cham: Springer International Publishing; 2020. pp. 608–621.

[52] Coogle CG, Storie S, Ottley JR, Rahn NL, Kurowski-Burt A. Technology-Enhanced Performance-Based Feedback to Support Teacher Practice and Child Outcomes. Topics in Early Childhood Special Education. 2021;41: 72–85. doi: 10.1177/0271121419838624

[53] Chu H-C, Chen J-M, Hwang G-J, Chen T-W. Effects of formative assessment in an augmented reality approach to conducting ubiquitous learning activities for architecture courses. Universal Access Inf. 2019;18: 221–230. doi: 10.1007/s10209-017-0588-y

[54] Zhang H, Magooda A, Litman D, Correnti R, Wang E, Matsmura LC, et al. eRevise: Using Natural Language Processing to Provide Formative Feedback on Text Evidence Usage in Student Writing. Proceedings of the AAAI Conference on Artificial Intelligence. 2019;33: 9619–9625.

[55] Edalati M, Imran AS, Kastrati Z, Daudpota SM. The Potential of Machine Learning Algorithms for Sentiment Classification of Students’ Feedback on MOOC. In: Arai K, editor. Intelligent Systems and Applications. Cham: Springer International Publishing; 2022. pp. 11–22.

[56] Zhang Y, Qian A, Pi Z, Yang J. Danmaku Related to Video Content Facilitates Learning. Journal of Educational Technology Systems. 2019;47: 359–372. doi: 10.1177/0047239518811933

[57] Chen Y, Gao Q, Yuan Q, Tang Y. Facilitating Students’ Interaction in MOOCs through Timeline-Anchored Discussion. International Journal of Human–Computer Interaction. 2019;35: 1781–1799. doi: 10.1080/10447318.2019.1574056

[58] Lin X, Huang M, Cordie L. An exploratory study: using Danmaku in online video-based lectures. Educational Media International. 2018;55: 273–286. doi: 10.1080/09523987.2018.1512447

[59] Fabriz S, Mendzheritskaya J, Stehle S. Impact of Synchronous and Asynchronous Settings of Online Teaching and Learning in Higher Education on Students’ Learning Experience During COVID-19. Frontiers in Psychology. 2021;12. doi: 10.3389/fpsyg.2021.733554 34707542 PMC8542673

[60] Ahmed MMH, McGahan PS, Indurkhya B, Kaneko K, Nakagawa M. Effects of synchronized and asynchronized e-feedback interactions on academic writing, achievement motivation and critical thinking. Knowledge Management & E-Learning: An International Journal. 2021;13: 290–315.

[61] Huffman S. Using Mobile Technologies for Synchronous Cmc to Develop L2 Oral Proficiency. Pronunciation in Second Language Learning and Teaching Proceedings. 2011;2. https://www.iastatedigitalpress.com/psllt/article/id/15165/.

[62] Payne JS, Whitney PJ. Developing L2 Oral Proficiency through Synchronous CMC: Output, Working Memory, and Interlanguage Development. CALICO Journal. 2002;20: 7–32. doi: 10.1558/cj.v20i1.7-32

[63] Creswell JW, Creswell JD. Research design: qualitative, quantitative, and mixed methods approaches. Fifth edition. Los Angeles: SAGE; 2018.

[64] Creswell JW, Plano Clark VL. Designing and conducting mixed methods research. Third Edition. Los Angeles: SAGE; 2018.

[65] Artieda G, Muñoz C. The LLAMA tests and the underlying structure of language aptitude at two levels of foreign language proficiency. Learning and Individual Differences. 2016;50: 42–48. doi: 10.1016/j.lindif.2016.06.023

[66] Li H. Are teachers teaching to the test? A case study of the College English Test (CET) in China. International Journal of Pedagogies and Learning. 2009;5: 25–36. doi: 10.5172/ijpl.5.1.25

[67] Charness G, Gneezy U, Kuhn MA. Experimental methods: Between-subject and within-subject design. Journal of Economic Behavior & Organization. 2012;81: 1–8. doi: 10.1016/j.jebo.2011.08.009

[68] Zhang Y, Elder C. Investigating native and non-native English-speaking teacher raters’ judgements of oral proficiency in the College English Test-Spoken English Test (CET-SET). Assessment in Education: Principles, Policy & Practice. 2014;21: 306–325. doi: 10.1080/0969594X.2013.845547

[69] Wu W-CV, Marek M, Chen N-S. Assessing cultural awareness and linguistic competency of EFL learners in a CMC-based active learning context. System. 2013;41: 515–528. doi: 10.1016/j.system.2013.05.004

[70] Birt L, Scott S, Cavers D, Campbell C, Walter F. Member Checking: A Tool to Enhance Trustworthiness or Merely a Nod to Validation? Qual Health Res. 2016;26: 1802–1811. doi: 10.1177/1049732316654870 27340178

[71] Conover WJ, Iman RL. Rank Transformations as a Bridge between Parametric and Nonparametric Statistics. The American Statistician. 1981;35: 124–129. doi: 10.1080/00031305.1981.10479327

[72] Braun V, Clarke V. Thematic analysis. APA handbook of research methods in psychology, Vol 2: Research designs: Quantitative, qualitative, neuropsychological, and biological. Washington, DC, US: American Psychological Association; 2012. pp. 57–71.

[73] Cascio MA, Lee E, Vaudrin N, Freedman DA. A Team-based Approach to Open Coding: Considerations for Creating Intercoder Consensus. Field Methods. 2019;31: 116–130. doi: 10.1177/1525822X19838237

[74] Nowell LS, Norris JM, White DE, Moules NJ. Thematic Analysis: Striving to Meet the Trustworthiness Criteria. Int J Qual Meth. 2017;16: 1609406917733847. doi: 10.1177/1609406917733847

[75] Archibald MM. Investigator triangulation: A collaborative strategy with potential for mixed methods research. Journal of Mixed Methods Research. 2016;10: 228–250. doi: 10.1177/1558689815570092

[76] Janesick VJ. Peer Debriefing. The Blackwell Encyclopedia of Sociology. John Wiley & Sons, Ltd; 2015.

[77] Kostova I. Thick Description. The Wiley-Blackwell Encyclopedia of Social Theory. John Wiley & Sons, Ltd; 2017. pp. 1–2.

[78] Al Jahromi D. Can Teacher and Peer Formative Feedback Enhance L2 University Students’ Oral Presentation Skills? In: Hidri S, editor. Changing Language Assessment: New Dimensions, New Challenges. Cham: Springer International Publishing; 2020. pp. 95–131.

[79] Wood J. Making peer feedback work: the contribution of technology-mediated dialogic peer feedback to feedback uptake and literacy. Assessment & Evaluation in Higher Education. 2022;47: 327–346. doi: 10.1080/02602938.2021.1914544

[80] Morley D, Carmichael H. Engagement in socio constructivist online learning to support personalisation and borderless education. Student Engagement in Higher Education Journal. 2020;3: 115–132. Available: https://sehej.raise-network.com/raise/article/view/1004.

[81] Rambe P. Activity theory and technology mediated interaction: Cognitive scaffolding using question-based consultation on Facebook. Australasian Journal of Educational Technology. 2012;28. doi: 10.14742/ajet.775

[82] Emmerson D. The use of synchronous and asynchronous technological tools for socio-constructivist language learning. jltl. 2019;9: 1–6. https://dergipark.org.tr/en/pub/jltl/issue/46605/555908.

[83] Lee S-M. Factors affecting incidental L2 vocabulary acquisition and retention in a game-enhanced learning environment. ReCALL. 2022; 1–16. doi: 10.1017/S0958344022000209

[84] Teng MF. Language Learning Through Captioned Videos. 1st edition. New York: Routledge; 2020.

[85] Elborolosy SAM. Using Drama Approach and Oral Corrective Feedback in Enhancing Language Intelligibility and Oral Fluency among English Majors. TPLS. 2020;10: 1453. doi: 10.17507/tpls.1011.16

[86] Weissheimer J, Caldas V, Marques F. Using Whatsapp to develop L2 oral production. Leitura. 2018; 21–38. doi: 10.28998/2317-9945.2018v1n60p21-38

[87] Qiu X, Cheng H. The effects of task types on L2 oral production and learner engagement. International Review of Applied Linguistics in Language Teaching. 2022;60: 1063–1088. doi: 10.1515/iral-2020-0128

[88] Hui YK, Li C, Qian S, Kwok LF. Enhancing students’ engagement by giving ongoing formative feedback in a blended learning setting. International Journal of Innovation and Learning. 2021;30: 390–407. doi: 10.1504/IJIL.2021.118190

[89] Seligman L, Abdullahi A, Teherani A, Hauer KE. From Grading to Assessment for Learning: A Qualitative Study of Student Perceptions Surrounding Elimination of Core Clerkship Grades and Enhanced Formative Feedback. Teaching and Learning in Medicine. 2021;33: 314–325. doi: 10.1080/10401334.2020.1847654 33228392

[90] Drews R, Tani G, Cardozo P, Chiviacowsky S. Positive feedback praising good performance does not alter the learning of an intrinsically motivating task in 10-year-old children. European Journal of Human Movement. 2020;45. doi: 10.21134/eurjhm.2020.45.5

[91] Scales PC, Pekel K, Sethi J, Chamberlain R, Van Boekel M. Academic Year Changes in Student-Teacher Developmental Relationships and Their Linkage to Middle and High School Students’ Motivation: A Mixed Methods Study. The Journal of Early Adolescence. 2020;40: 499–536. doi: 10.1177/0272431619858414

[92] Moran J, Briscoe G, Peglow S. Current Technology in Advancing Medical Education: Perspectives for Learning and Providing Care. Acad Psychiatry. 2018;42: 796–799. doi: 10.1007/s40596-018-0946-y 29949053

[93] Yuan R, Mak P. Reflective learning and identity construction in practice, discourse and activity: Experiences of pre-service language teachers in Hong Kong. Teaching and Teacher Education. 2018;74: 205–214. doi: 10.1016/j.tate.2018.05.009

[94] Colomer J, Serra T, Cañabate D, Bubnys R. Reflective Learning in Higher Education: Active Methodologies for Transformative Practices. Sustainability. 2020;12: 3827. doi: 10.3390/su12093827

[95] Henderson M, Phillips M. Video-based feedback on student assessment: scarily personal. Australasian Journal of Educational Technology. 2015;31. doi: 10.14742/ajet.1878

